# The Shark Alar Hypothalamus: Molecular Characterization of Prosomeric Subdivisions and Evolutionary Trends

**DOI:** 10.3389/fnana.2016.00113

**Published:** 2016-11-24

**Authors:** Gabriel N. Santos-Durán, Susana Ferreiro-Galve, Arnaud Menuet, Idoia Quintana-Urzainqui, Sylvie Mazan, Isabel Rodríguez-Moldes, Eva Candal

**Affiliations:** ^1^Grupo BRAINSHARK, Departamento de Biología Funcional, Universidade de Santiago de CompostelaSantiago de Compostela, Spain; ^2^CNRS, UMR 7355, University of OrleansOrleans, France; ^3^Centre for Integrative Physiology, University of EdinburghEdinburgh, UK; ^4^Sorbonne Universités, UPMC, CNRS UMR7232 Biologie Intégrative des Organismes Marins, Observatoire OcéanologiqueBanyuls sur Mer, France

**Keywords:** chondrichthyan, forebrain, alar/basal boundary, evolution, development, *Pax6*, *Otp*, *Dlx*

## Abstract

The hypothalamus is an important physiologic center of the vertebrate brain involved in the elaboration of individual and species survival responses. To better understand the ancestral organization of the alar hypothalamus we revisit previous data on *ScOtp*, *ScDlx2/5*, *ScTbr1*, *ScNkx2.*1 expression and Pax6 immunoreactivity jointly with new data on *ScNeurog2*, *ScLhx9*, *ScLhx5*, and *ScNkx2.8* expression, in addition to immunoreactivity to serotonin (5-HT) and doublecortin (DCX) in the catshark *Scyliorhinus canicula*, a key species for this purpose since cartilaginous fishes are basal representatives of gnathostomes (jawed vertebrates). Our study revealed a complex genoarchitecture for the chondrichthyan alar hypothalamus. We identified terminal (rostral) and peduncular (caudal) subdivisions in the prosomeric paraventricular and subparaventricular areas (TPa/PPa and TSPa/PSPa, respectively) evidenced by the expression pattern of developmental genes like *ScLhx5* (TPa) and immunoreactivity against Pax6 (PSPa) and 5-HT (PPa and PSPa). Dorso-ventral subdivisions were only evidenced in the SPa (SPaD, SPaV; respectively) by means of Pax6 and *ScNkx2.8* (respectively). Interestingly, *ScNkx2.8* expression overlaps over the alar-basal boundary, as *Nkx2.2* does in other vertebrates. Our results reveal evidences for the existence of different groups of tangentially migrated cells expressing *ScOtp*, *Pax6*, and *ScDlx2*. The genoarchitectonic comparative analysis suggests alternative interpretations of the rostral-most alar plate in prosomeric terms and reveals a conserved molecular background for the vertebrate alar hypothalamus likely acquired before/during the agnathan-gnathostome transition, on which *Otp*, *Pax6*, *Lhx5*, and *Neurog2* are expressed in the Pa while *Dlx* and *Nkx2.2/Nkx2.*8 are expressed in the SPa.

## Introduction

The hypothalamus is a conserved integrative center that coordinates autonomic, endocrine, and limbic responses. Its organization is the result of complex patterning processes that converge at the rostral-most part of the neural tube, which in turn, yields a complex structure that has been difficult to systematize (Shimamura et al., 1995; Puelles and Rubenstein, 2003, 2015; Puelles et al., 2004, 2012; Medina, 2008; Szabó et al., 2009; Shimogori et al., 2010; Alvarez-Bolado et al., 2012, 2015; Croizier et al., 2015). As a result, its development has been recently a topic of active research (Alvarez-Bolado et al., 2015).

Classically, the mammalian hypothalamus has been described as consisting of a wide variety of neuronal clusters subdivided in four regions: preoptic, anterior, tuberal, and mamillary (Simerly, 2004). This organization relies on conceptions that understood the brain to be organized in functional columns (also referred as “columnar models”), in which the hypothalamus would be located ventrally to the remaining diencephalon. Alternative brain conceptions have understood the brain to be divided in transverse segments (or neuromeres) yielding segmental (or neuromeric) paradigms of brain organization (Puelles, 2009). Modern segmental paradigms, such as the “prosomeric model,” recognize the mentioned hypothalamic regions under an alternative axial concept that relies on known developmental processes and in neuromeric organization (Puelles and Rubenstein, 2003, 2015; Puelles, 2009; Puelles et al., 2012). Furthermore, the prosomeric model also considers that these segments are organized in different histogenetic territories defined by neuroepithelial transcription factor specification codes and radial units (Puelles et al., 2012; Nieuwenhuys and Puelles, 2016). This paradigm understands the hypothalamus to be ventrally located with respect to the telencephalon (thus, rostral to the remaining diencephalon) forming together a pair of hypothalamo-telencephalic segmental units (hp1, hp2) at the rostral-most part of the neural tube. Under these criteria the preoptic area is recognized as part of the subpallial telencephalon rather than belonging to the hypothalamus (Flames et al., 2007; Medina and Abellán, 2012; Puelles et al., 2012).

The updated prosomeric model recognizes the hypothalamus to be subdivided in dorso-ventrally arranged histogenetic domains. The intrahypothalamic border (IHB) divides these histogenetic domains in rostral (hp2 or terminal) and caudal (hp1 or peduncular) portions. As a result, in the alar plate the hypothalamus presents at least four progenitor subdomains (from rostral to caudal and dorsal to ventral): terminal and peduncular paraventricular area (TPa, PPa; respectively), terminal and peduncular subparaventricular area (TSPa, PSPa; respectively). Further dorso-ventral subdivisions have also been proposed (Morales-Delgado et al., 2011, 2014; Puelles et al., 2012; Díaz et al., 2015; Ferrán et al., 2015; Puelles and Rubenstein, 2015). The relationships among these domains and hypothalamic nuclei have been pointed out: the TPa/PPa domain gives rise to magnocellular and parvocellular neurosecretory populations of the supraopto-paraventricular complex; the TSPa/PSPa domain will form mainly the suprachiasmatic nucleus, the anterior hypothalamic nucleus, and the subparaventricular zone, while the basal hypothalamus gives rise to classical tuberal and mamillary derivatives (Morales-Delgado et al., 2011; Puelles et al., 2012; Herget et al., 2014; Herget and Ryu, 2015). Finally, additional histogenic domains are recognized in the acroterminal territory, the rostral-most portion of the neural tube (see Puelles et al., 2012; Ferrán et al., 2015; Puelles and Rubenstein, 2015; Santos-Durán et al., 2015). This median region is responsible of the development of structures such as the *lamina terminalis* or the optic chiasm in the alar hypothalamus.

Because of their phylogenetic position as out-group to osteichthyans (the other major phylum of gnathostomes, which includes bony fish and tetrapods), chondrichthyans are essential to reconstruct gnathostome ancestral characteristics through comparisons with other vertebrate models. Recently, we have carried out a preliminary study of the molecular histogenetic organization of the hypothalamus of an elasmobranch representative, the catshark *Scyliorhinus canicula*, and we analyzed this organization under the updated prosomeric framework (see Santos-Durán et al., 2015). Such analysis revealed a strikingly high degree in the conservation of hypothalamic histogenetic compartments between chondrichthyan and murine models. Indeed, the alar expression of *ScOtp* and *ScDlx2/5* revealed apparently conserved paraventricular (Pa) and subparaventricular (SPa) progenitor domains. The basal expression of these and other genes lead to the identification of tuberal/retrotuberal (Tu/RTu), perimamillary/periretromamillary (PM/PRM) and mamillary/retromamillary (MM/RM) domains, apparently homologous to those described in murine models. Besides, a molecular hypothalamo-telencephalic border (HTB) and a hypothalamo-diencephalic border (HDB), matching with those described in the model, were identified in the shark, together with an IHB defined, as in mouse (Puelles et al., 2012), based on the course of ascending tracts to the telencephalon (see Santos-Durán et al., 2015).

In such previous study some histogenetic differences were also observed within particular subdomains, but their significance has not been explored so far. Moreover, although many of the boundaries and assumptions predicted by the prosomeric model were confirmed in the chondrichthyan model, further dorso-ventral subdivisions and genetic evidences of rostro-caudal segmentation, particularly concerning the alar hypothalamus, have not been previously addressed. Besides, the meaning of the results observed in the shark remains to be analyzed in evolutionary context. For all these reasons, here we have examined more deeply the molecular profile of the alar hypothalamus of *S. canicula*, with three aims: (i) to look for further prosomeric molecular subdivisions, (ii) to better define the molecular alar-basal boundary (ABB) and (iii) to obtain some insights on the evolution of the alar hypothalamus by comparative analysis. To address these questions, previous data on *ScOtp, ScDlx2/5*, *ScTbr1*, *ScNkx2.1* expression and Pax6 immunoreactivity were revised jointly with new data on *ScNeurog2*, *ScLhx9*, *ScLhx5*, and *ScNkx2.8* expression and serotonin (5-HT) and doublecortin (DCX) immunoreactivity. Further details on the rostro-caudal and dorso-ventral molecular organization of the alar hypothalamus obtained with these genes support partially the existence of subdomains similar to those proposed by the prosomeric model. Particularly, terminal and peduncular subdivisions were defined in the Pa and SPa domains, but a dorsoventral subdivision only could be distinguished in the SPa domain.

## Materials and Methods

### Experimental Animals

Some embryos of the catshark (also known as the lesser spotted dogfish; *S. canicula*) were supplied by the Marine Biological Model Supply Service of the CNRS UPMC Roscoff Biological Station (France) and the Estación de Bioloxía Mariña da Graña of the University of Santiago de Compostela. Additional embryos were kindly provided by the Aquaria of Gijón (Asturias, Spain), O Grove (Pontevedra, Spain) and Finisterrae (A Coruña, Spain). Embryos were staged by their external features according to Ballard et al. (1993). For more information about the relationship of the embryonic stages with body size, gestation and birth, see Table 1 in Ferreiro-Galve et al. (2010). Fifty embryos from stages 18 to 32 were used in this study. Eggs from different broods were raised in seawater tanks in standard conditions of temperature (15–16°C), pH (7.5–8.5) and salinity (35 g/L). Adequate measures were taken to minimize animal pain or discomfort. All procedures conformed to the guidelines established by the European Communities Council Directive of 22 September 2010 (2010/63/UE) and by the Spanish Royal Decree 53/2013 for animal experimentation and were approved by the Ethics Committee of the University of Santiago de Compostela.

### Tissue Processing

Embryos were deeply anesthetized with 0.5% tricaine methane sulfonate (MS-222; Sigma, St. Louis, MO, USA) in sea water and separated from the yolk before fixation in 4% Paraformaldehyde (PFA) in elasmobranch’s phosphate buffered [EPB: 0.1 M phosphate buffer (PB) containing 1,75% urea, pH 7.4] for 48–72 h depending on the stage of development. Subsequently, they were rinsed in phosphate buffered saline (PBS), cryoprotected with 30% sucrose in PB, embedded in OCT compound (Tissue Tek, Torrance, CA, USA), and frozen with liquid nitrogen-cooled isopentane. Parallel series of sections (12–20 μm thick) were obtained in transverse planes on a cryostat and mounted on Superfrost Plus (Menzel-Glasser, Madison, WI, USA) slides.

### Single Immunohistochemistry (IHC) on Sections

For heat-induced epitope retrieval, sections were pre-treated with 0.01 M citrate buffer (pH 6.0) for 30 min at 95°C and allowed to cool for 20–30 min at room temperature (RT). Sections were then rinsed twice in 0.05 M Tris-buffer saline (TBS; pH 7.4) for 5 min each and incubated overnight with the primary antibody (rabbit anti-Pax6 polyclonal antiserum, Covance, Emeryville, CA, USA, diluted 1:400; polyclonal rabbit anti-Sonic Hedgehog [anti-Shh], Santa Cruz Biotechnology, Santa Cruz, CA, USA, diluted 1:300; polyclonal rabbit anti-doublecortin [anti-DCX] Cell Signaling; diluted 1:300–500; rabbit anti-serotonin [anti-5-HT] polyclonal antiserum, DiaSorin, Immunostar, Hudson, WI, USA, diluted 1:5000). Appropriate secondary antibody (horseradish peroxidase [HRP])-conjugated goat anti-rabbit were incubated for 2 h at RT. For immunofluorescence, appropriate secondary antibody was used (DAR546 [Alexa 546-conjugated donkey anti-rabbit] Molecular Probes, Eugene, OR, USA, diluted 1:100). Sections were rinsed in distilled water (twice for 30 min), allowed to dry for 2 h at 37°C and mounted in MOWIOL 4-88 Reagent (Calbiochem, MerkKGaA, Darmstadt, Germany). All dilutions were made with TBS containing 15% donkey normal serum (DNS; Millipore, Billerica, MA, USA), 0.2% Triton X-100 (Sigma) and 2% bovine serum albumin (BSA, Sigma).

### Controls and Specificity of the Antibodies

No immunostaining was detected when primary or secondary antibodies were omitted during incubations. For details about the specificity of anti-5-HT and anti-DCX antibodies, see Pose-Méndez et al. (2014). For details about the specificity of anti-Pax6 antibody, see Quintana-Urzainqui et al. (2014). The polyclonal anti-Shh antibody (Santa Cruz Biotechnology, Inc., Santa Cruz, CA, USA) was raised in rabbit against the amino acids 41–200 of Shh human protein. The *in situ* hybridization (ISH) results were similar to those obtained by immunohistochemistry (IHC), and therefore validate the specificity of the anti-Shh antibody used here.

### *In situ* Hybridization (ISH) on Sections and Whole Mounts Embryos

We applied ISH for *ScOtp* (Santos-Durán et al., 2015), *ScDlx2* (Quintana-Urzainqui et al., 2012, 2015; Compagnucci et al., 2013; Debiais-Thibaud et al., 2013; Santos-Durán et al., 2015), *ScDlx5* (Compagnucci et al., 2013; Debiais-Thibaud et al., 2013; Santos-Durán et al., 2015), *ScLhx9* (Pose-Méndez et al., 2015; Quintana-Urzainqui et al., 2015), *ScLhx5, ScNkx2* (Quintana-Urzainqui et al., 2012, 2015; Santos-Durán et al., 2015), *ScNkx2.8, ScTbr1* (Quintana-Urzainqui et al., 2015; Santos-Durán et al., 2015), and *ScNeurog2* genes. These probes were selected from a collection of *S. canicula* embryonic cDNA library (mixed stages S9–S22), constructed in pSPORT1, and submitted to high throughput EST sequencing. cDNA fragments were cloned in pSPORT vectors. Sense and antisense digoxigenin-UTP-labeled and fluorescein-UTP-labeled probes were synthesized directly by *in vitro* transcription using as templates linearized recombinant plasmid DNA or cDNA fragments prepared by PCR amplification of the recombinant plasmids. ISH in whole mount and on cryostat sections was carried out following standard protocols (Coolen et al., 2009). Briefly, sections were permeabilized with proteinase K, hybridized with sense or antisense probes overnight at 65°C and incubated with the alkaline phosphatase-coupled anti-digoxigenin and anti-fluorescein antibody (1:2000, Roche Applied Science, Manheim, Germany) overnight at 4°C. The color reaction was performed in the presence of BM-Purple (Roche). Control sense probes did not produce any detectable signal. Immunohistochemistry was performed after ISH to obtain double ISH-IHC staining as described above.

#### Inhibition of the Shh Pathway

Inhibition of the Shh pathway was performed by *in ovo* injection of the pharmacological inhibitor cyclopamine to test if the initiation of *ScNkx2.8* expression in the forebrain, specifically in the ABB, is dependent on Shh, similarly to other *Nkx* paralogs like *Nkx2.1* or *Nkx2.2*. First, 200 μL of a solution containing 1X PBS, 500 μM cyclopamine and 5% dimethyl sulfoxide (DMSO) were injected through the shell of stage 15–16 *S. canicula* eggs. This solution was replaced by the same volume of 5% DMSO in 1X PBS for control embryos. The eggs were maintained for 3 days in oxygenated sea water at 17°C, with viabilities higher than 90%. Embryos reached stage 18 in these conditions. They were dissected, fixed in PFA 4%, dehydrated and stored in methanol 100% prior to ISH.

#### Image Acquisition and Analysis

Light field images were obtained with an Olympus BX51 microscope equipped with an Olympus DP71 color digital camera. Fluorescent sections were photographed with an epifluorescence photomicroscope Olympus AX70 fitted with an Olympus DP70 color digital camera. Photographs were adjusted for brightness and contrast and plates were prepared using Adobe Photoshop CS4 (Adobe, San Jose, CA, USA).

## Results

Genes whose expressions define broader domains (*ScOtp* and *ScDlx2/5*) are described first and different subdomains are defined by analyzing the Pax6 immunoreactivity pattern and the expression pattern of the following genes: *ScTbr1*, *ScNeurog2*, *ScLhx9*, *ScLhx5*, *ScNkx2.1*, *ScNkx2.8*.

### *ScOtp* Expression

An overview of the expression of *ScOtp* in the hypothalamus of *S. canicula* from early to late stages of development has been previously reported (Santos-Durán et al., 2015). In the alar plate *ScOtp* is mainly expressed in the Pa territory, and therefore it is helpful in recognizing this histogenetic domain. To deepen in the genoarchitectonic profile of this compartment, in this study we have checked the expression of *ScOtp* mainly by the analysis of transverse sections through the hypothalamus of embryos from stage 29 to 32.

**FIGURE 1:**
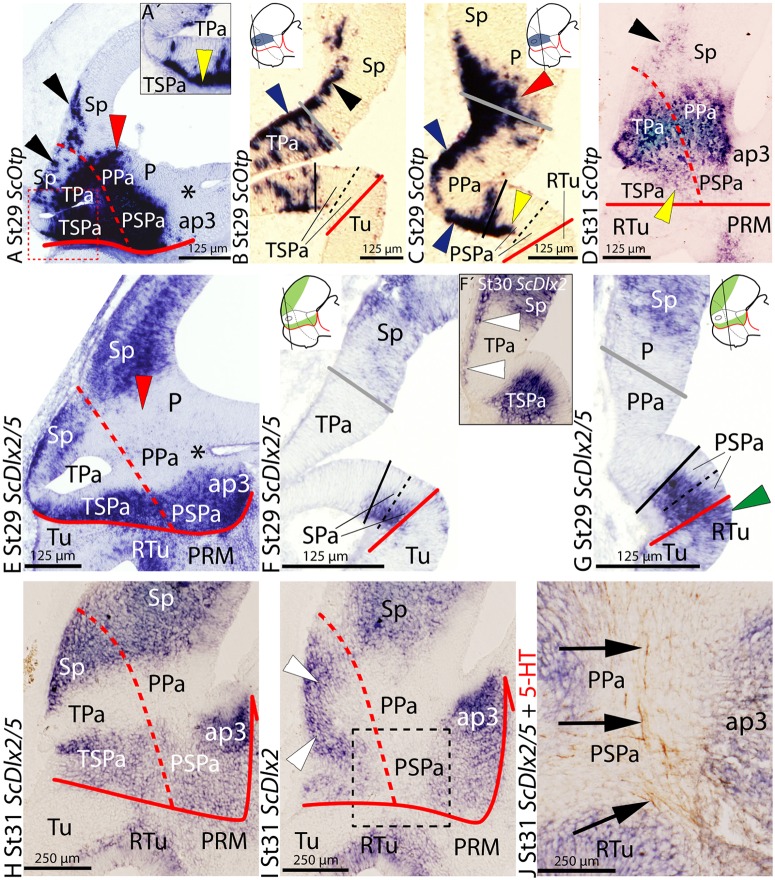
**Regionalization of the alar hypothalamus and neighbor territories in embryos of *Scyliorhinus canicula* at stages 29–31 based on the expression of *ScOtp*** **(A–D)** and *ScDlx2/5* **(E–J)** expression by means of single *in situ* hybridization (ISH) **(A–J)** and/or combined with immunohistochemistry **(J)** on sagittal **(A,D,E,H–J)** or transverse **(B,C,F,G)** sections. Continuous red line: ABB. Dashed red line: IHB. Gray line: HTB. Continuous black line divides dorso-ventral division of the alar hypothalamus into Pa (dorsal) and SPa (ventral) territories. Dashed black line represents subdivisions inside the SPa into SPa dorsal (SPaD) and ventral (SPaV). Asterisk point the PThE (ap3). **(A–D)** *ScOtp* expression in the Pa at indicated stages. *ScOtp* labeling (yellow arrowheads) in the SPa corresponds to marginal cells. Black arrowheads point to *ScOtp*-expressing cells in the subpallium. Red arrowheads point to *ScOtp*-expressing cells in the pallium. Blue arrowheads point to marginal *ScOtp*-expressing cells in the Pa. Yellow arrowheads point to *ScOtp*-expressing cells ventral to the Pa. **(A′)** Detail of a region equivalent to that squared area in **(A)** to show *ScOtp*-expressing cells ventral to the Pa rostrally. **(E–J)** *ScDlx2/5* expression in the subpallium, SPa and ap3 at indicated stages. Red arrowhead points to the lack of expression in the pallium. White arrowheads point to *ScDlx2*-expressing **(F′)** cells in the marginal zone of the Pa. Green arrowhead in **(G)** points to *ScDlx2/5* expression in the RTu. **(J)** Detail of a region equivalent to that squared in **(I)**. Arrows point to 5-HT-ir tracts coursing in the marginal Pa and SPa. For abbreviations, see list.

At stage 29, *ScOtp* is expressed in the surroundings of the optic stalk and caudally beyond, in what mainly represents the Pa histogenetic domain, as seen in sagittal (**Figure 1A**) and transverse (**Figures 1B,C**) sections. *ScOtp* is recognized in individual cells in the TPa domain (**Figure 1B**) mostly located in the marginal zone (blue arrowhead in **Figure 1B**) but also scattered through the ventricular zone. Similarly, in the PPa domain (**Figure 1C**), *ScOtp* is mainly expressed in the marginal zone (blue arrowheads in **Figure 1C**), while scarce *ScOtp*-expressing cells are observed in the ventricular zone. Furthermore, *ScOtp*-expressing cells are recognized in the marginal zone of territories placed dorsal and ventral with respect to the TPa/PPa domain, forming a continuous stream with the marginal *ScOtp*-expressing cells of this domain. Particularly noticeable are the strings of *ScOtp*-expressing cells dorsally extended from the TPa domain into the subpallial territory (black arrowheads in **Figures 1A,B**) and from the rostral-most portion of the PPa domain into the pallium (red arrowheads in **Figures 1A,C**). *ScOtp*-expressing cells are also observed in the marginal zone ventral to the TPa/PPa domain. These cells cannot be observed at the rostral-most TSPa domain (**Figure 1B**) but they spread just caudal from this point into the remaining TSPa/PSPa domain (yellow arrowheads in **Figures 1A′,C**). Of note, these ventral *ScOtp*-expressing cells are distributed into the dorsal-most marginal zone of TSPa/PSPa domains (yellow arrowhead in **Figure 1C**) but they spread through the SPa domain by late stage 29 (not shown).

This basic pattern is maintained until later stages of development (**Figure 1D**) with minor modifications. *ScOtp* is abundantly expressed in radial domains extending from the ventricular to the marginal zone at stage 32 and also in juveniles (data not shown).

### *ScDlx2*/*ScDlx5* Expression

The expression of *ScDlx5* from stage 18 onward and the expression of *ScDlx2* from stage 29 onward have been previously reported in Santos-Durán et al. (2015). Essentially identical results were observed with both genes in the brain of *S. canicula* from stage 29 onward, so we use *ScDlx2/5* at these stages to refer indistinctly to both patterns. However, differences are also observed which will be commented where appropriate (see below). Here, we reexamine these data to additionally analyze the expression of *ScDlx2/5* from stage 29 to 32. We further characterize the ABB and possible dorso-ventral and rostro-caudal subdomains of the alar hypothalamus, mainly by the analysis of transverse sections through the hypothalamus.

At stage 29, in the alar plate of the secondary prosencephalon and rostral diencephalon, *ScDlx2/5* is expressed in the subpallium, the SPa domain, and in the prethalamus, while it is also expressed in some subdivision in the basal plate subdivisions (**Figures 1E–G**; see also Santos-Durán et al., 2015). Between the expression domains of *ScDlx2/5* in the subpallium and SPa territory there is a negative wedge-shaped domain spreading from the optic stalk to the pallium that contains the territory of the Pa compartment and the adjacent diencephalic region that corresponds to the prethalamic eminence (PThE), the dorsal-most part of the alar prosomere 3 (asterisk in **Figure 1E**; see also Santos-Durán et al., 2015). On transverse sections, the expression of *ScDlx2/5* in the SPa compartment can be recognized ventrally to the optic stalk (**Figure 1F**). Interestingly, in the subpallium, *ScDlx2/5* expression occupies the whole ventricular wall while in the SPa domain it does not (**Figures 1F,G**). In the PSPa compartment, the expression of *ScDlx2/5* expands ventrally into domains of the basal hypothalamus (green arrowhead in **Figure 1G**) although the extent of the ventricular expression differs between both territories. The ventricular expression of *ScDlx2/5* between the SPa domain and rostral diencephalon also differs (data not shown).

Noteworthy, from stage 29 onward, *ScDlx2-* but not *ScDlx5*-expressing cells are observed in the marginal zone of the TPa domain (white arrowheads in **Figure 1F′**), which is negative for *ScDlx5* expression. These cells form a continuous string with the positive *ScDlx2/5* domain in the subpallium.

This pattern is maintained until stage 31 (compare **Figures 1H,E**). At this stage, marginal *ScDlx2*-expressing cells form a continuous stripe between the subpallium and the TSPa domain (white arrowheads in **Figure 1I**). In parasagittal sections, 5-HT-immunoreactive (ir) fibers can be recognized ascending to the telencephalon through the alar peduncular hypothalamus (**Figure 1J**; Carrera et al., 2008). From this stage onward, *ScDlx2/5* expression is recognizable in the subventricular zone of the SPa (data not shown). At stage 32, *ScDlx2/5* expression is maintained both in the alar and basal hypothalamus (data not shown).

### *ScTbr1* Expression

*ScTbr1* expression was described at stage 25 in Santos-Durán et al. (2015), revealing that, though *ScTbr1* is not expressed in any region of the alar hypothalamus of *S. canicula* at such stage, it is a useful gene to define its boundaries. Here, we examine the detailed expression of *ScTbr1* from stage 29 to 32 in order to know if such usefulness remains throughout development.

**FIGURE 2:**
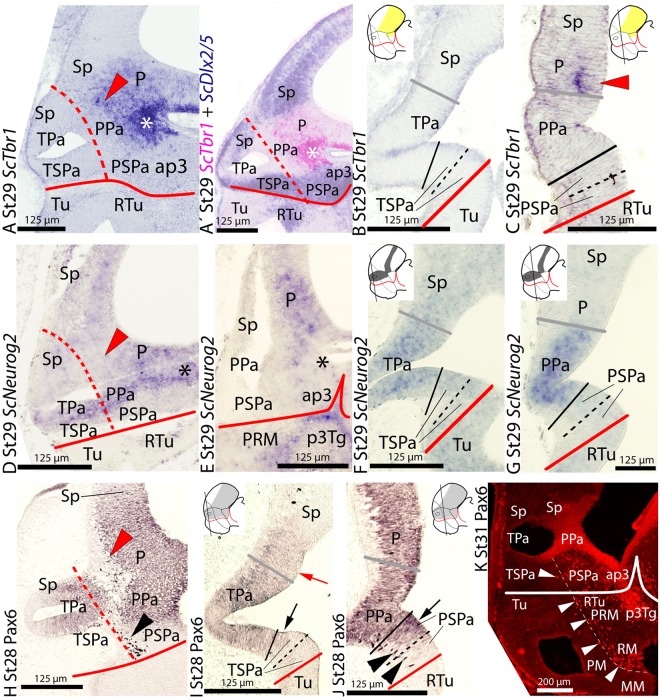
**Regionalization of the alar hypothalamus and neighbor territories in embryos of *S. canicula* at stages 28–31 based on the expression of *ScTbr1*** **(A–C)**, *ScDlx2/5* **(A′)** *ScNeurog2* **(D–G)** and Pax6 immunoreactivity **(H–K)** by means of single *ISH* on sections **(A–G)** and single immunoenzyme staining **(H–J)** or immunofluorescence **(K)** on sagittal **(A,D,E,H,K)** or transverse **(B,C,F,G,I,J)** sections. **(A–C)** *ScTbr1* expression in the pallium and PThE (asterisk). Red arrowhead points to rostral-most expression in the pallium. **(A′)** Image results from the overlapping of two parallel sections hybridized with *ScTbr1* and *ScDlx2/5* probes, respectively. Color for *ScTbr1* was digitally converted to pink to ease comparison. The complementary expression of *ScTbr1* and *ScDlx2/5* defines the Pa domain. **(D–G)** *ScNeurog2* expression in the Pa and pallium. Note that Figures **(D,E)** present artifacts corresponding to broken tissue. Red arrowhead points to the rostral-most pallium lacking *ScNeurog2*. **(H–K)** Pax6 immunoreactivity at indicated stages. Red arrowhead points to marginal Pax6 immunoreactivity at the rostral-most pallium. Black and white arrowheads point to marginal Pax6-ir cells in the peduncular SPa (PSPa) and PBHy. Arrows point to Pax6 immunoreactivity dorsal and ventral to the Pa. Note that the stream of Pax6-ir cells in **(K)** closely follows the IHB. For other labels, see legend in **Figure 1**. For abbreviations, see list.

At stage 29, *ScTbr1* continues to be expressed in the pallium and the adjacent territory of the rostral alar diencephalon, the PThE (**Figures 2A–C**; see also Figure 6B in Santos-Durán et al., 2015). Such expression abuts the dorsal and caudal border of the PPa area but not the TPa domain (**Figures 2A–C**; also compare red arrowheads in **Figures 2A,C** and **1A,C**). Its expression is roughly complementary to that of *ScDlx2/5* in the telencephalon and rostral diencephalon (**Figure 2A′**; also compare **Figures 2A,C** with **1E,G**; see also Figure 6B in Santos-Durán et al., 2015). Note that the expression of both genes, in turn, is complementary to the ventricular domains of *ScOtp* in the TPa/PPa area (**Figure 2A′**; also compare **Figures 2A**, **1E**, with **1A**). Together, the distinct but complementary expression of *ScTbr1*, *ScDlx2/5*, and *ScOtp* domains allow to define the whole alar plate of the secondary prosencephalon and rostral diencephalon, consistently with the prosomeric model.

From stage 31 this pattern is maintained in the alar hypothalamus, and the changes observed in the telencephalon were already reported (see Quintana-Urzainqui et al., 2015).

### *ScNeurog2* Expression

At stage 29, *ScNeurog2* is expressed in restricted domains of the alar and basal plates of the prosencephalon and rostral diencephalon (**Figures 2D–G**). In the alar plate, *ScNeurog2* is expressed in the ventricular zone of the hypothalamus (TPa/PPa domains; **Figures 2D–G**), as well as in the pallium and the PThE. *ScNeurog2* is less intensely expressed in the TPa than in the PPa (compare **Figures 2F,G**). *ScNeurog2* expression matches the ventricular domains of *ScOtp* in the TPa/PPa domain (compare **Figures 2D–G** with **Figures 1A–C**) and abuts the *ScDlx2/5*-expressing TSPa/PSPa domain (compare **Figures 2D–G** with **Figures 1E–G**). At odds with *ScOtp*, *ScNeurog2*-expressing cells are not observed in the marginal zones of the TPa/PPa domain or surrounding territories. This basic pattern is maintained until stage 30. At stage 31, *ScNeurog2* is downregulated in the alar hypothalamus although it is maintained in the pallium (not shown).

### Pax6 Immunoreactivity

In the shark, the basic segmental organization of the forebrain was examined from mid-gestation to late stages of development based on Pax6 immunoreactivity by Ferreiro-Galve et al. (2008). Besides, *ScPax6* expression, which matched Pax6 immunoreactivity, was analyzed in the whole brain from early to late stages of development by Ferreiro-Galve (2010). However, none of these studies was undertaken using the updated prosomeric model (in fact, the alar hypothalamus as such was not considered in those studies). Here, we revisited these data in the context of the updated prosomeric model from stage 28 until stage 32.

At stage 28, Pax6 immunoreactivity is observed in the ventricular zone of a domain continuous between the alar hypothalamus and both the telencephalic pallium (**Figures 2H–J**) and the rostral diencephalon including the PThE (data not shown). Pax6 immunoreaction rather contrasts with the *ScDlx2/5*-expressing TSPa/PSPa domain (compare **Figure 2H** with **Figure 1E**), though some overlapping exists between both developmental genes at ventricular levels in the dorsal and ventral part of this domain (see black arrows in **Figures 2I,J**). Besides, Pax6-ir cells are observed in the marginal zone of a restricted area of the PSPa domain (black arrowheads in **Figures 2H,J**).

From stage 30 onward, marginal Pax6-ir cells are abundant throughout the diencephalon, including the basal plate, tegmental areas (**Figure 2K**; see also Ferreiro-Galve et al., 2008), as well as in the peduncular alar and basal hypothalamus; in contrast, they are largely absent at the terminal hypothalamus, thus emphasizing the IHB (**Figure 2K**; see also IHB in Figure 6B in Santos-Durán et al., 2015).

This basic pattern is maintained until late stages of development but Pax6 immunoreactivity is intense in cells that populate the mantle zone of alar and basal plates in the prosencephalon. As previously reported, they are especially abundant in the PPa area, rostral diencephalon and pallium (Ferreiro-Galve et al., 2008; Rodríguez-Moldes, 2009; Quintana-Urzainqui et al., 2015).

### *ScLhx9* Expression

**FIGURE 3:**
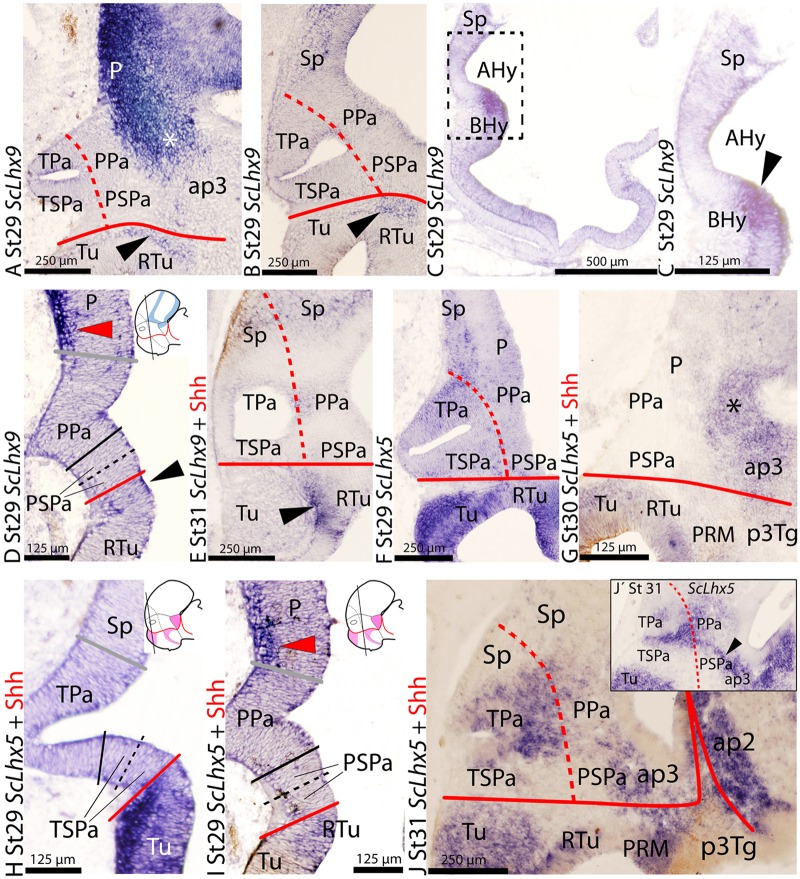
**Regionalization of the alar hypothalamus and/or neighbor territories in embryos of *S. canicula* at stages 29–31 based on the expression of *ScLhx9* (A–E)**, and *ScLhx5* **(F–J)** on sagittal **(A–C′,E–G,J)** or transverse **(D,H,I)** sections. Some of them were double labeled for immunohistochemistry against Shh **(E,G,H–J)**. **(A–E)** *ScLhx9* expression in the limits of the alar hypothalamus, pallium, PThE (asterisk) and basal hypothalamus, at indicated stages. Black arrowhead points to *ScLhx9* expression in the basal hypothalamus just ventral to the SPa. Red arrowhead points to *ScLhx9*-expressing cells in the pallium. **(C)** Section medial to **(B)**. **(C′)** Detail of the squared region in **(C)** showing absence of *ScLhx9* expression from the rostral-most alar hypothalamus. **(F–J)** *ScLhx5*-expression in different regions of the prosencephalon including the alar hypothalamus. **(I)** Red arrowhead points to *ScLhx5*-expressing cells in the pallium. **(J′)** Sagittal section lateral to **(J)**. Black arrowhead points to a string of *ScLhx5*-expressing cells extending along the alar hypothalamus (PPa) and ap3. For other labels, see legend **Figure 1**. For abbreviations, see list.

*ScLhx9* expression has been previously reported in the meso-isthmo-cerebellar region of *S. canicula* from stage 25 to stage 27 (Pose-Méndez et al., 2015) and in the pallium (medial, ventral) of embryos from stage 31 onward (Quintana-Urzainqui et al., 2015). Here, we report that at early stages (25–28), *ScLhx9* is also expressed in restricted domains of the secondary prosencephalon and rostral diencephalon. From stage 29 onward, *ScLhx9* is also expressed in restricted domains of the secondary prosencephalon and rostral diencephalon, i.e., it is expressed dorsal, ventral and caudal to the alar hypothalamus but not in it, as can be seen in sagittal (**Figures 3A–C′,E**) and transverse sections through the hypothalamus (**Figure 3D**). These territories include ventricular and/or *ScLhx9*-expressing marginal cells in restricted areas of the pallium and rostral diencephalon (**Figure 3A**), subpallium and basal hypothalamus (**Figure 3B**).

In the alar plate, *ScLhx9*-expressing cells are only observed in the marginal zone of the pallium and the rostral diencephalon (compare **Figures 3A,D**). In the basal hypothalamus, *ScLhx9* is expressed in a restricted longitudinal and dorsal domain that abuts the PSPa domain (black arrowheads in **Figures 3A,B,D**).

This basic pattern is maintained until late stages of development (**Figure 3E**) with minor modifications concerning marginal *ScLhx9*-expressing cells in the pallium (see also Quintana-Urzainqui et al., 2015).

### *ScLhx5* Expression

At stage 29, the earliest we have studied, *ScLhx5* is expressed in many different restricted domains of the alar and basal plates of the prosencephalon including pallium, subpallium, alar and basal hypothalamus and rostral diencephalon, as observed in sagittal (**Figures 3F,G**) and transverse (**Figures 3H,I**) sections through the hypothalamus.

In the alar plate of stage 29 and 30 specimens, *ScLhx5*-expressing cells are observed in the marginal zone of pallial territories dorsal to the PPa, similar to *ScLhx9* pattern (red arrowhead in **Figure 3I**; compare with *ScLhx9* in **Figure 3D** and also with *ScTbr1* expression in **Figure 2C**). From this stage onward, *ScLhx5* is also expressed in the rostral diencephalon (**Figure 3G**).

At stage 31 (**Figures 3J,J′**), *ScLhx5* is expressed in the subventricular zone and mantle of the Pa domain. Of note, it is more extensively expressed in the TPa territory than in the PPa. However in the PPa territory, a noticeable string of cells continuous with that of the rostral diencephalon is observed (black arrowhead in **Figure 3J′**). At stage 32, intense *ScLhx5* signal was observed only in the telencephalon while it decreases in the hypothalamus (data not shown).

### *ScNkx2.1* Expression

*ScNkx2.1* expression and its comparison with that of *Shh* were previously reported from early stages of development up to stage 29 in Santos-Durán et al. (2015). Though both genes are not observed in the alar hypothalamus, they are useful to determine boundaries with neighbor territories of the basal hypothalamus and/or the subpallium. Here, we have additionally analyzed the expression of *ScNkx2.1* from stage 29 to 32 to further characterize the ABB later in development and the possible dorso-ventral and rostro-caudal subdomains of the alar hypothalamus, mainly by the analysis of transverse sections through the hypothalamus. With the same aim, we have comparatively analyzed *ScNkx2.1* expression and Shh immunoreactivity at stage 29.

**FIGURE 4:**
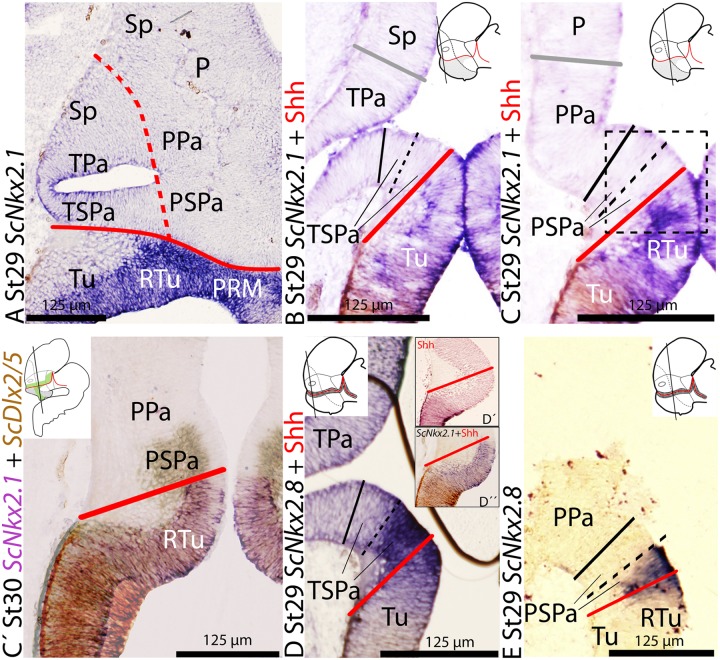
**Regionalization of the alar hypothalamus and neighbor territories in embryos of *S. canicula* at stages 29–30 based on the expression of *ScNkx2.1*** **(A–C,D″)** and *ScNkx2.8* **(D,E)** on sagittal **(A)** and transverse **(B,E)** sections. Some sections were labeled **(D′)** or double labeled for immunohistochemistry against Shh **(B–D′)**. **(C′)** Detail of the squared regions in Figure **(C)**. It results from the overlapping of two parallel sections hybridized with *ScDlx2/5*and*ScNkx2.1* probes, respectively. Color for *ScDlx2/5* and *ScNkx2.1* was digitally converted to brown and purple to ease comparison. **(A–C)** *ScNkx2.1* expression at indicated stages. **(D,E)** *ScNkx2.8* expression at indicated stages. Note that Shh immunoreactivity does not reach the ABB **(D′,D″**). For other labels, see legend **Figure 1**. For abbreviations, see list.

At stage 29, *ScNkx2.1* is expressed ventral to the optic stalk through the basal hypothalamus except in the RM compartment (not shown), forming a sharp limit with the ABB (see continuous red line in **Figures 4A–C**; see also Santos-Durán et al., 2015). Furthermore, *ScNkx2.1* and Shh immunoreactivity co-distribute in the Tu region (**Figures 4B,C**), though Shh immunoreactivity does not match the dorsal border of *ScNkx2.1* expression. Notably, the dorsal border of *ScNkx2.1* appears to abut the ventral border of *ScDlx2/5* in the TSPa/PSPa area (**Figure 4C′**). Dorsal to the alar hypothalamus, *ScNkx2.1* expression and Shh immunoreactivity also co-distribute in the preoptic subpallium (data not shown; see Quintana-Urzainqui et al., 2012, 2015). Both genes abut the dorsal limit of the alar hypothalamus (**Figures 4A,B**).

This basic pattern is maintained until late stages of development.

### *ScNkx2.8* Expression

The expression of *Nkx2.8* ortholog has not been described during brain development of other vertebrates. However, its expression pattern fairly coincides with that of other genes overlapping over the ABB such as *Nkx2.2* or *Nkx2.9* (Puelles et al., 2012). To characterize *ScNkx2.8* expression here we analyzed the expression patterns of *ScNkx2.8* through development and, particularly, its expression at stage 25 combined with anti-doublecortin (DCX) immunohistochemistry to identify pioneering tracts.

The expression of *ScNkx2.8* was analyzed from stage 18 until midgestation of development in supplemental material (see **Supplementary Figure S2**). From stage 28 onward, two dorso-ventrally arranged subdomains can be differentiated throughout the longitudinal expression of *ScNkx2.8*, the dorsal subdomain being more intense than the ventral one (see continuous red line in **Figures 4D,E**). *ScNkx2.8* expression is observed ventral to the optic stalk (**Figures 4D,E**), overlapping the territory where the basal expression of *ScNkx2.1* appears to abut the alar expression of *ScDlx2/5* (see **Figure 4C′**). The dorsal intense domain is expressed in the ventral-most TSPa/PSPa domain or supraliminal alar area (**Figures 4D,E**). The ventral less intense domain corresponds to the dorsal-most Tu/RTu area or subliminal basal area (**Figures 4D,E**). Furthermore, Shh immunoreactivity abuts the ventral portion of *ScNkx2.8* but not the ABB (**Figures 4D–D″**), at least at ventricular levels.

The mentioned pattern is maintained at later stages of development. However, the expression in the forebrain becomes downregulated (although still recognizable) from stage 31 onward (data not shown).

## Discussion

We have identified terminal (rostral) and peduncular (caudal) subdivisions in the prosomeric paraventricular and subparaventricular areas by analyzing the expression pattern of various developmental genes (*ScOtp*, *ScDlx2/5*, *ScTbr1*, *ScNkx2.1*, *ScNeurog2*, *ScLhx9*, *ScLhx5*, and *ScNkx2.8*) and immunoreactivity against molecular markers such as Pax6, 5-HT and DCX. While we are aware that those molecular markers do not define *per se* histological domains, their expressions have been described in homologous regions in different species, and then, they have been largely shown to be useful in the attempt of recognizing particular domains. However, the functional role of many of the genes considered and the biological significance of their expressions are not always deeply understood and thus we cannot discard that their expression dynamics in late embryos could not account for different subdomains but rather for functions unrelated to patterning.

### Alar-Basal Boundary

The ABB is an important landmark continuously defined in the prosomeric model (Puelles and Rubenstein, 1993, 2003, 2015; Puelles et al., 2012). In mice, this linear forebrain boundary is defined between the early Pax6-positive alar plate and the *Shh*- and *Nkx2.1*-positive basal plate (Puelles et al., 2012). Therefore, it is a virtual or lineal boundary located between the plasma membrane of cells located in alar and basal plates. Moreover, it is well-known that the gene *Nkx2.2* (and other genes co-expressing with it such as *Nkx2.9*, *Ptc* and *Gsx*) overlap over this boundary being expressed in restricted domains within alar and basal plates (Puelles et al., 2012).

**FIGURE 5:**
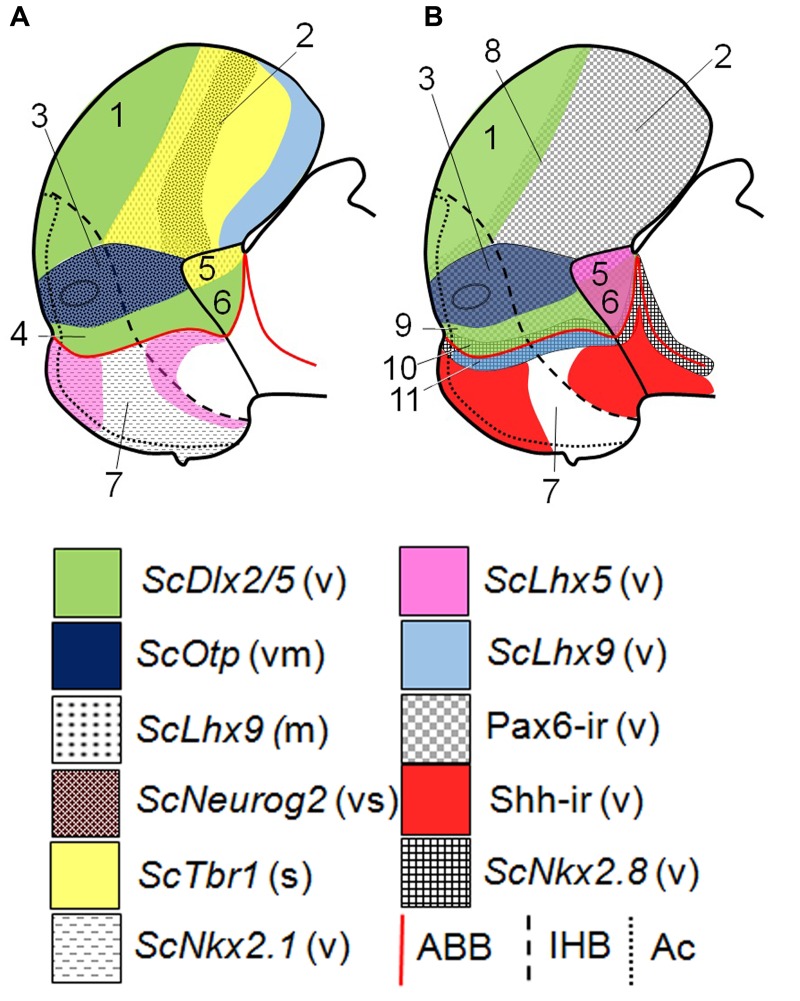
**Schematic representation of various gene expressions in the telencephalon, hypothalamus and rostral diencephalon of *S. canicula* at stage 29.** **(A)** Domains and subdomains defined by *ScDlx2/5, ScOtp*, *ScLhx9, ScNeurog2*, *ScTbr1*, and *ScNkx2.1* expression patterns. Some of these genes define subdivisions in the pallium. **(B)** Domains and subdomains defined by *ScDlx2/5, ScOtp*, *ScLhx9*, *ScLhx5*, Pax6 immunoreactivity, Shh immunoreactivity and *ScNkx2.8* expression. Continuous red line represents ABB. Discontinuous black line represents IHB. Dotted line limits acroterminal region. Domains: (1) Subpallium, (2) Pallium, (3) Pa, (4) SPa, (5) PThE, (6) Alar p3, (7) Basal hypothalamus, (8) Pallium-Subpallium boundary, (9) SPaD, (10) SPaV (or supraliminal), (11) SIBHy (or subliminal). For abbreviations, see list.

Although, in a previous work we defined the shark ABB mainly based on the expression of *ScShh* (Santos-Durán et al., 2015), our present data made us to reconsider this boundary since we found that *ScDlx2/5* and *ScNkx2.1* were the unique pairs of alar-basal markers that abut at a certain point (**Figures 5A,B**). Moreover, as described for *Nkx2.2* in other vertebrates, we found that *ScNkx2.8* overlap the virtual line defined by *ScDlx2/5* and *ScNkx2.1* (**Figures 5A,B**). Furthermore, in the alar plate, *ScNkx2.8* abuts Pax6 immunoreactivity dorsally and co-distributes with *ScDlx2/5* (**Figure 5B**) while in the basal plate, *ScNkx2.8* co-distributes with *ScNkx2.1* and abuts Shh immunoreactivity ventrally (**Figure 5B**), invalidating our previous definition. Therefore, we think that in the shark the abutted expression of *ScDlx2/5-ScNkx2.1*, which can also be followed by the expression of *ScNkx2.8* in a band overlapping this linear boundary, represents best the ABB.

Although more evidences coming from studies in other vertebrates are needed, the following observations in shark support that the *Nkx2.8* ortholog is likely expressed over the ABB: (i) *ScNkx2.8* expression closely resembles the pattern of *Nkx2.2* in other vertebrates over the ABB (Barth and Wilson, 1995; Sugahara et al., 2011; Puelles et al., 2012; Ware et al., 2014; Domínguez et al., 2015), (ii) it co-distributes with the course of the tract of the postoptic commisure (TPOC), which is also assumed to overlap the ABB (see **Supplementary Figures S1D,D′**; Barth and Wilson, 1995; Shimamura et al., 1995; Puelles et al., 2012; Ware et al., 2014); (iii) it is expressed partly in the alar plate and partly in the basal plate, as shown by *Nkx2.2* in the mouse (see **Figures 4D,E**; see also Puelles et al., 2012; Ware et al., 2014; Puelles and Rubenstein, 2015); and (iv) its expression is induced by the Shh pathway (see **Supplementary Figures S2A,B**) as other paralogs of *Nkx* family in the forebrain (see Santos-Durán et al., 2015).

### Prosomeric Compartments and Subcompartments of the Alar Hypothalamus

Based on the alar complementary expression of *ScOtp* and *ScDlx2/5*, we have previously identified the shark alar hypothalamus harboring a Pa and SPa domains and tentatively defined its rostro-caudal subdivision by the course of the medial forebrain bundle (mfb) through the rostral border of hp1, just caudal to the optic stalk (Santos-Durán et al., 2015). However, we did not find molecular evidence for further rostro-caudal or dorso-ventral subdivisions. Here, we deepen in the genoarchitectonic profile of the shark alar hypothalamus providing molecular insights of their dorso-ventral and rostro-caudal subdivisions, and further analyzing histogenetic implications of the genes expressed. Noteworthy, in the stages considered, the developmental control genes here studied did not reveal further details of the acroterminal territory although the updated prosomeric model (Puelles et al., 2012; Ferrán et al., 2015) proposes that the optic stalk and the optic chiasm belong to acroterminal subdivisions of the Pa and SPa, respectively.

#### Pa: Genoarchitectonic Profile and Further Subdivisions

**FIGURE 6:**
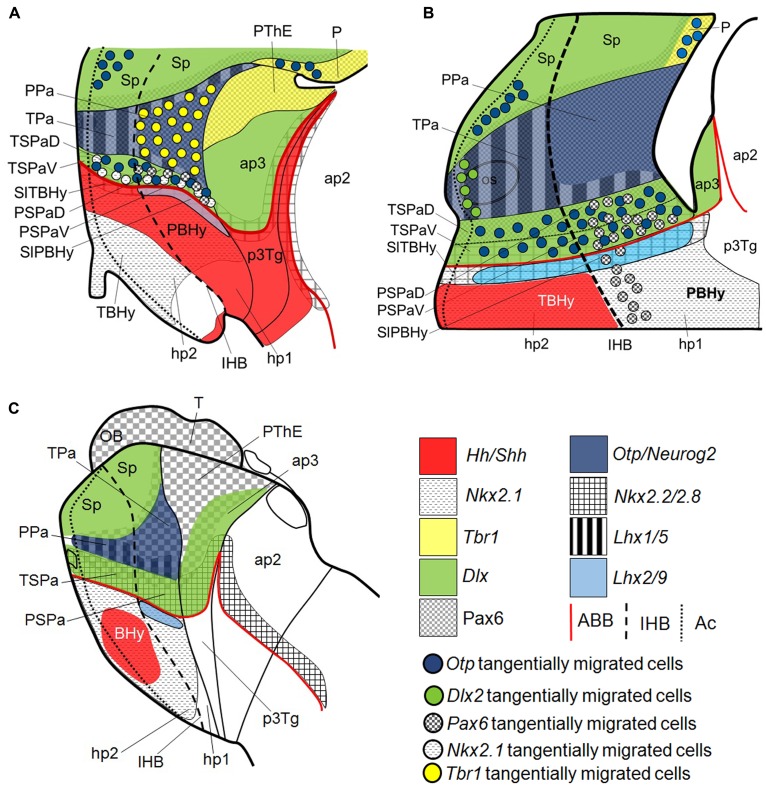
**Schematic representation of various gene expression patterns in the telencephalon, hypothalamus and rostral diencephalon of (A)** developing mouse adapted from Puelles et al. (2012); **(B)** *S. canicula* at stage 31; **(C)** lamprey adapted from Martínez-de-la-Torre et al. (2011). Domains and subdomains defined by *Otp*, *Dlx2*, *Pax6*, *Neurog2*, *Lhx9*, *Lhx5*, *Tbr1*, *Nkx2.8*, *Nkx2.2*, *Shh*, *Hh*, and *Nkx2.1* orthologs. Continuous red line represents ABB. Discontinuous black line represents IHB. Dotted line limits acroterminal region. For abbreviations, see list.

The updated prosomeric model defined the Pa as a domain characterized by the expression of *Otp* and the lack of *Dlx2/5* (Morales-Delgado et al., 2011; Puelles et al., 2012; Puelles and Rubenstein, 2015). In this territory, *Otp* expression co-distributes with that of *Pax6*, *Neurog2*, *Lhx5* or *Tbr1* genes (Stoykova et al., 1996; Medina, 2008; Abellán et al., 2010; Osório et al., 2010; Puelles et al., 2012; Ferrán et al., 2015) among others. Restricted expression patterns and certain progenitor subdomains led the authors of the model to divide the Pa into terminal or peduncular (**Figure 6A**; TPa, PPa; respectively) and different dorso-ventral subdomains (Morales-Delgado et al., 2011, 2014; Puelles et al., 2012; Ferrán et al., 2015; Puelles and Rubenstein, 2015). Moreover, the course of ascending tracts to the telencephalon through the rostral-most portion of the peduncular hypothalamus supports the existence of rostro-caudal subdivisions (Puelles et al., 2012; Puelles and Rubenstein, 2015).

We have previously characterized the shark Pa domain by the expression of *ScOtp* and we distinguished its rostro-caudal subdivisions by the course of the mfb caudal to the rostral border of hp1 (Santos-Durán et al., 2015). Here we show that in this domain, similarly to what was reported in mice and other tetrapods (Stoykova et al., 1996; Medina, 2008; Abellán et al., 2010; Osório et al., 2010; Puelles et al., 2012; Domínguez et al., 2013, 2015), *ScOtp* co-distributes with Pax6, *ScNeurog2* and *ScLhx5* (see **Figures 5A,B**). We have not found evidences supporting dorso-ventral subdivision. Terminal-peduncular subdomains can be sketched by the restricted expression of *ScLhx5*, but not based on *Tbr1*-expressing cells, as in mice (**Figure 6A**; Puelles et al., 2012; Ferrán et al., 2015), neither on *Nkx2.2* expression, as suggested in other vertebrates (Domínguez et al., 2015). In the shark, from stage 30 onward, *ScLhx5* seems to be more abundantly expressed in the TPa than in the PPa (**Figure 6B**). It forms an evident border (see **Figures 3J,J′**; see also **Figure 6B**) located rostrally with respect to the 5-HT-ir tracts that course along the mfb (compare **Figures 3J,J′** with **1J**) in hp1. These facts support the existence of molecular rostro-caudal differences in the Pa territory (**Figure 6B**; see also Figure 6B in Santos-Durán et al., 2015; see also Puelles et al., 2012) but also of the IHB as suggested by the prosomeric model. These data also evidence a striking high degree of conservation between the expression patterns found in chondrichthyans and mammals.

#### SPa: Further Prosomeric Subdivisions

The updated prosomeric model defined the SPa as a domain characterized by the expression of *Dlx2/5* adjacent to the ABB (Morales-Delgado et al., 2011; Puelles et al., 2012; Puelles and Rubenstein, 2015). Gene markers like *Lhx1*, *Nkx2.1* or *Nkx2.2*, among others are co-expressed with *Dlx2/5* in this territory (Shimogori et al., 2010; Puelles et al., 2012; Ferrán et al., 2015). As for the Pa, restricted expression patterns and other evidences suggest that the SPa has a rostro-caudal (terminal TSPa and peduncular PSP) and a dorso-ventral (dorsal SPa [SPaD] and ventral SPa [SPaV] or supraliminal) regionalization (Morales-Delgado et al., 2011; Puelles et al., 2012; Díaz et al., 2015; see also **Figure 5B**).

In the shark, we have previously identified the SPa domain by the expression of *ScDlx2/5*, the lack of *ScOtp*, and characterized its rostro-caudal subdivisions by the course of the mfb caudal to the rostral border of hp1 (Santos-Durán et al., 2015). Our present results reveal that Pax6, *ScNkx2.8* and *ScOtp* co-distribute with *ScDlx2/5* in the SPa (**Figure 6B**). However, these makers are not expressed homogenously through this area, which lead us to recognize four microzones as predicted by the prosomeric model (Morales-Delgado et al., 2011; Puelles et al., 2012; Puelles and Rubenstein, 2015).

A SPaD can be defined by the co-distribution of *ScDlx2/5* and Pax6, the latter extending ventralward beyond Pa into SPaD (see continuous and dashed black lines in **Figures 1F,G** and **2I,J**, respectively; see also **Figure 6B**). In this subdomain, Pax6 immunoreactivity is not detected through the whole ventricular surface and characteristically shows a weak labeling (see black arrow in **Figures 2I,J**; see also **Figure 6B**), which appears to be common in regions where *Pax6* and *Dlx* co-distribute (like the pallium-subpallium boundary; see below). Moreover, a SPaV (or supraliminal) territory can be defined in shark by the ventricular co-distribution of *ScDlx2/5* and *ScNkx2.8* (compare **Figures 1F,G** with **4H,I**; see also **Figures 5B** and **6B**). As discussed above, *ScNkx2.8* overlaps the ABB, being expressed in the alar and basal plates. So it abuts Pax6 immunoreactivity dorsally (compare **Figures 4H,I** with **2I,J**; see also **Figures 5B** and **6B**) and Shh immunoreactivity ventrally in the rostral basal hypothalamus (**Figures 4D,D′**; see also **Figures 5B** and **6B**). Taking these data together, the dorso-ventral compartmentalization in the shark SPa territory seems evident, but slight differences with respect to that described in mammals are noted, especially in the SPaV compartment (compare **Figures 6A,B**). Moreover, a rostro-caudal regionalization based on the differential distribution of presumed tangentially migrated cells is consistent with the model proposal of terminal (TSPa) and peduncular (PSPa) subdomains by the intersection of dorso-ventral and rostro-caudal differences (TSPaD and TSPaV; PSPaD, and PSPaV, respectively; see below).

#### Tangential Migrations Involving the SPa and Basal Hypothalamus

In the mouse, tangential migrations involving the hypothalamus as source and/or recipient territory have been characterized by means of immunohistochemistry, autoradiography and ISH techniques (Morales-Delgado et al., 2011; Puelles et al., 2012; Morales-Delgado et al., 2014; Croizier et al., 2015; Díaz et al., 2015). The study of some of these intra-hypothalamic migrations led to the identification of three dorso-ventral progenitor subdomains (with their respective rostro-caudal subdivisions) in the Pa of the mouse (Morales-Delgado et al., 2011, 2014; Puelles et al., 2012). Furthermore, tangentially migrated cells derived from the Pa have been also described as contributing to extra-hypothalamic territories such as the amygdala (García-Moreno et al., 2010).

Although further studies using cell-tracking/tracing techniques should be needed to confirm it, in the present study we have identified putative pathways of tangential migrations based on the spatio-temporal patterns of different genes widely used to identify tangentially migrating neurons, including cells expressing *ScDlx2*, *ScOtp* and cells showing Pax6 immunoreactivity. Although, these cells seem to involve the hypothalamus as source or recipient territories, here, we consider those restricted inside the hypothalamic territory leading us to further characterize the SPa but not the Pa domain. Because the specification process that leads to the expression of these genes is different from those of the recipient territories, we interpret that these cells in fact could have migrated tangentially as also suggested by Morales-Delgado et al. (2011) in mouse.

In the Pa territory, *ScOtp* expression is continuous with *ScOtp*-expressing cells that spread into the marginal zone of the SPaD region (see yellow arrowheads in **Figures 1A′,C,D**; see also **Figure 6B**) excepting at the rostral-most portion of the TSPa region (see **Figure 1B**) a fact compatible with the idea of acroterminal subdivisions (Ferrán et al., 2015). However, by late stage 29 *ScOtp*-expressing cells are observed in the subventricular zone of the *ScDlx2/5*-positive SPaD/SPaV domain (data not shown). Taking into account their spatio-temporal distribution, as argued above, we consider that such *Otp*-expressing cells may have migrated tangentially from the Pa to the SPa territories. Similar *Otp*-positive cells have been also observed in the Spa region of the mouse (Bardet et al., 2008; Morales-Delgado et al., 2011). Moreover, *Otp* has been involved in the development of the catecholaminergic phenotype in the zebrafish hypothalamus (Del Giacco et al., 2006, 2008; Blechman et al., 2007; Ryu et al., 2007), suggesting a correlation among Sc*Otp*-expressing cells and TH-ir cells in the GAD-ir SPa area of the shark (see Figure 1A in Ferreiro-Galve et al., 2008; note that the alar hypothalamus is not described in prosomeric terms).

Furthermore, a rostro-caudal regionalization in the SPa domain can be distinguished based on cells showing Pax6 immunoreactivity presumed to be tangentially migrated cells. These cells are observed in the mantle zone of Pax6- and *ScDlx2/5*-positive domains in the PSPaD territory (black arrowheads in **Figures 2H,J**; compare with **Figures 1F,G**; see also **Figure 6B**). Similar Pax6-positive cells can be observed at the pallial-subpallial boundary, in the mantle of the dorsal lateral ganglionic eminence (LGE) and other *Dlx* territories pointing that the presence of Pax6-ir cells in the mantle is common in regions where *Pax6* and *Dlx* co-distribute (Puelles et al., 2000; Flames et al., 2007; Ferreiro-Galve et al., 2008; Medina et al., 2011; Quintana-Urzainqui et al., 2015). Although at stage 28, these cells seem to occupy both the PSPaD and the PSPaV territories (**Figure 2J**) they are only observed caudal to the TSPa domain forming a continuous stream with diencephalic marginal Pax6-ir cells (black arrowheads in **Figures 2H,J**; agreeing with previous descriptions in Ferreiro-Galve (2010) although using a different terminology). Notably, similar cells have been found in the mouse addressed to the “posterior entopeduncular area” which parsimoniously fit with the updated hp1 prosomere (see **Figures 6A,B**) (Stoykova et al., 1996; Puelles et al., 2012). From stage 30 onward these cells spread into the hypothalamic basal plate (white arrowheads in **Figure 2K**) forming a striking stream that closely abuts the IHB recognized in Santos-Durán et al., 2015). Besides, these cells apparently reach the MM/RM border, and thus, the hp2/hp1 limit (Puelles et al., 2012; Santos-Durán et al., 2015), which again fits with the model. Thus we interpret that these Pax6-ir cells are caudal to the IHB likely accompanying tracts coursing by the rostral border of hp1 (of note, the updated model provided an explanatory framework for this observation). In fact, Pax6 expression has been involved in the correct pathfinding of different tract systems through the prosencephalon by means of both, indirect and local mechanisms, which supports this idea (Mastick et al., 1997; Vitalis et al., 2000; Jones et al., 2002; Prestoz et al., 2012). Taking together these observations, we interpret that TSPa can be differentiated from PSPa based on these cells both in the mouse and the shark (see **Figure 6B**; see also Stoykova et al., 1996; Puelles et al., 2012).

Finally, we also have observed a group of *ScDlx2*-expressing cells continuous with the subpallium in the marginal zone of the TPa region (arrows **Figure 1F′**). As discussed above, since *Dlx* and *Otp* expression are the result of different developmental processes we interpret that these cells migrate tangentially. Of note, at later stages and in an equivalent position, *ScDlx2*-expressing cells form a continuous string between the *ScDlx2/5* expressing subpallium and the TSPa domain suggesting the existence of a telencephalic-hypothalamic stream (arrowheads in **Figure 1I**; see **Figure 6B**). Interestingly, Tripodi et al. (2004) and Shimogori et al. (2010) respectively detected COUP-TF-expressing and *Foxg1*-expressing cells likely emanating from the ventral telencephalon into the anterior hypothalamus. Furthermore, one of the migrating streams described by Tripodi et al. (2004) seems to course from the subpallium to the suprachiasmatic region (equivalent to the SPa) through the marginal anterior hypothalamus (equivalent to the Pa) resembling what we observed in the shark (see their Figure 6B). Thus, the existence of a tangential telencephalic-hypothalamic stream could be conserved among vertebrates similarly to other migrations that have been recently identified in the telencephalon of the shark (Quintana-Urzainqui et al., 2015).

#### Alternative Interpretations of the Alar Hypothalamus

In a previous work, we defined the HTB based on the complementary expression of *ScOtp* and *ScFoxg1a* and the HDB based on the complementary expression of *ScOtp* and *ScTbr1* (see Santos-Durán et al., 2015). Here, we show that additional developmental control genes can also define these borders. The abutted expression of *ScOtp* in the hypothalamus with *ScDlx2/5* in the subpallium and *ScTbr1*/*ScLhx9* in the pallium outlines the HTB (**Figures 5A,B**). The HDB can be also sketched based on the expression of *ScOtp* in the hypothalamus abutting *ScLhx9* or *ScLhx5* in the alar p3 (**Figures 5A,B**). These results further support the genoarchitectonic subdivisions proposed by the prosomeric model.

However, an alternative interpretation of these data could yield different conclusions concerning the HTB, the HDB and the genoarchitectonic organization of the alar and rostral-most prosencephalon. Firstly, although the abutted expression of *Foxg1*/*Otp* is assumed to define the HTB, *Foxg1* has also been described beyond the telencephalic stalk (Pratt et al., 2004; Affaticati et al., 2015; Santos-Durán et al., 2015) questioning its telencephalon-restricted identity and thus the existence of the HTB at this place. Besides, in *Foxg1* null mutant mice the ventral, but not the dorsal, telencephalon is lost (Xuan et al., 1995; Martynoga et al., 2005), which suggests it is required for the development of the subpallium but maybe not for the whole telencephalon.

In this line of thought, the continuous ventricular expression of *Pax6* and *Neurog2* through the alar hypothalamus, pallium and rostral diencephalon (**Figure 5A**; see also Stoykova et al., 1996; Osório et al., 2010) also questions the expression of *Foxg1* defining the HTB and also the HDB. Strikingly, the fact that in the updated prosomeric model neither the Pa nor the HTB reach the HDB at the roof plate (see Figure 8.5B in Puelles et al., 2012), implicitly supports a continuity between hypothalamus, pallium and rostral diencephalon.

Besides, having taken into account data from mouse lacking neural Shh (Szabó et al., 2009), the continuous expression of *Lhx5* through the Pa-rostral diencephalon (PThE) and the continuous lack of *Dlx2* through the SPa-rostral diencephalon (excepting PThE) suggest: (i) a continuum between the alar hypothalamus and the alar rostral diencephalon; (ii) a dorso-ventral organization of this territory into Pax6 (dorsal) and *Dlx2* (ventral) domains; and (iii) the lack of the HDB.

Together, these evidences suggest an alternative interpretation of the alar prosencephalon (up to the zli) organized in a Pax6-ir domain (Pa, pallium and PThE) separating two *Dlx*-expressing domains (subpallium, and SPa besides the remaining rostral-most diencephalon; see **Figure 5A**) without a HTB and a HDB. Noteworthy, recently Affaticati et al. (2015) propose the orr (optic recess region) as a morphogenetic entity including *Dlx2* subdomains dorsal and ventral to *Otp* expression in the alar hypothalamus. These *Dlx*-expresing subdomains likely co-distribute with Pax6 as has been described in the pallium-subpallium boundary (Flames et al., 2007; Ferreiro-Galve et al., 2008) and in the SPaD (present results) pointing to *Pax6* as an important factor patterning the alar and rostral-most prosencephalon. Nevertheless, *Pax6* downregulation in the Pa territory of amphibians (Moreno et al., 2008) contradicts these observations although it could be an acquired trait since it is likely expressed in other anamniotes (see below). However, our unorthodox proposal should be considered as an open question to be pursued further in future studies.

### Evo-Devo Considerations Concerning the Alar Hypothalamus

Our analysis reveals that the data described for the chondrychthyan model, *S. canicula*, globally fit with the general assumptions and further details of the prosomeric model together with an important part of the data described in mouse (see above; see also Puelles et al., 2012; Santos-Durán et al., 2015). These facts support the early acquisition of these traits in development and evolution, which explain the similarities observed across vertebrates at a certain level of analysis, being also the base of the establishment of homologies (Puelles and Medina, 2002; Nieuwenhuys and Puelles, 2016). Differences are also observed but they mainly correspond to terminal phenotype and not the organization (rostro-caudal; ventro-dorsal) of subcompartments, which could explain local differences in proliferation and differentiation patterns across organisms, yielding different morphologies and neuronal subtypes.

To better understand the evolutionary meaning of these observations, here we have comparatively reviewed our findings with data described in other vertebrates. A similar analysis was made by other authors in the context of precedent prosomeric conceptions (Wullimann et al., 2005; van den Akker et al., 2008; Osório et al., 2010; Moreno and González, 2011; Moreno et al., 2012; Domínguez et al., 2013) and a recent work reviewed data on the anamniote-amniote transition under the updated paradigm (Domínguez et al., 2015). The analysis of our data in shark, based on comparing the presence/absence of expression of certain genes in equivalent topological regions, disagree with some of their interpretations concerning some acquisitions at the anamniote-amniote transition. To shed light on this matter, we have performed a further review of available results in bony fishes and agnathans to better know the anamniote scenario. We are aware of the difficulties to carry out such comparison (mainly because of the scarce number of detailed works specifically addressing the alar hypothalamus, the misleading hypothalamic nomenclature found in the literature, or interpretative differences of the model among authors, to name some).

#### Vertebrate Pa

The available information about genes expressed in the Pa of vertebrates as mouse, chick, *Xenopus*, zebrafish and lamprey reveals the following scenario: *Otp* is suggested to be expressed in the Pa territory of all the vertebrates studied so far, *Lhx5* seems to be expressed only in tetrapods, and *Pax6* and *Neurog2* seem to be an amniote acquisition (Frowein et al., 2002; Wullimann et al., 2005; Joly et al., 2007; Osório et al., 2010; Moreno and González, 2011; Moreno et al., 2012; Domínguez et al., 2013, 2015). Our present data in a shark representative of basal gnathostomes support the early acquisition of *Otp* and also reveal an acquisition of *Neurog2*, *Pax6* and *Lhx5* earlier than previously suggested.

Our data clearly support that the expression of *Lhx5* is present in early gnathostomes besides mammals (Shimogori et al., 2010), birds (Abellán et al., 2010), and amphibians (see “rostral supraoptoparaventricular area” in Domínguez et al., 2013, 2015). Moreover, *Lhx5* expression has been also described in the alar hypothalamus of other fishes (see “preoptic supraoptoparaventricular area” in Manoli et al., 2014). However, its expression appears to be already present in agnathans since *Lhx1/5* has been clearly described in the magnocellular preoptic nucleus of *Petromyzon marinus* (Osório et al., 2006) and *Lampetra fluviatilis* (see Figure 5B in Osorio et al., 2005). Taking together these observations, we interpret that, likely, *Lhx5*-like expression was early acquired in the Pa domain of vertebrates.

Pax6 has been described in the alar hypothalamus of amniotes (Li et al., 1994; Stoykova and Gruss, 1994; Stoykova et al., 1996; Puelles et al., 2000; Moreno et al., 2012; Robertshaw et al., 2013 and their supplemental material) but not in *Xenopus* (Bachy et al., 2002; Moreno et al., 2008). The expression of Pax6 in shark (Ferreiro-Galve et al., 2008; present results) supports an early acquisition in the alar hypothalamus of basal gnathostomes. The distribution of Pax6 data in zebrafish suggest that its distribution in the caudal-most preoptic area (Wullimann and Rink, 2001; Wullimann and Mueller, 2004), likely corresponds to the updated Pa domain and, similarly, the distribution of cells showing Pax6 immunoreactivity in the trout *Salmo trutta fario* (unpublished observations) is compatible with this idea. In lampreys, *Pax6* is likely to be expressed in the Pa domain (**Figure 6C**) (Murakami et al., 2001; Derobert et al., 2002; Uchida et al., 2003; Osorio et al., 2005; Sugahara et al., 2011; Suzuki et al., 2015). Its expression has not been necessarily described in the alar hypothalamus but can be recognized by the abutted (dorsal and ventral) expression of *Dlx1/6* (see Murakami et al., 2001). Moreover, the developing SOT (equivalent to the mfb as suggested by Puelles et al., 2012) divide the mentioned Pax6 domain in rostral and caudal portions (see Figures 2B and 4C2,C3 in Suzuki et al., 2015; for more data concerning the course of this tract in lamprey see Barreiro-Iglesias et al., 2008), as described for the shark (present results). Sugahara et al. (2011) also demonstrated that *Pax6* expression in the rostral secondary prosencephalon of *Lethenteron japonicum* seems to be regulated by the *Shh* and *Fgf* pathways. Noteworthy, the inhibition of such pathways performed by Sugahara et al. (2011) yield a *Pax6* expression pattern similar to that found in *Lampetra japonica* by Murakami et al. (2001) (compare Figures 5E and 7E in the first, with 7E in the second). Thus, *Pax6* seems to be acquired but differentially expressed in lampreys. Together, all these data point that a Pax6 expressing Pa domain seems to be present in early vertebrates being an early conserved trait of vertebrates.

Members of the *neurogenin* family have been reported in the forebrain of different vertebrate groups, including lampreys (Wullimann et al., 2005; Guérin et al., 2009; Nieber et al., 2009; Osório et al., 2010; Robertshaw et al., 2013). *Neurog2* has been described in the pallium, prethalamic eminence and Pa territory of amniotes (see “supraoptic/paraventricular” and anterior hypothalamus in Medina et al., 2004; Osório et al., 2010; see also Puelles et al., 2012). In chick, there are no detailed data though it has been described in an equivalent region to the Pa domain during a short developmental window (see Robertshaw et al., 2013, and their supplemental material). In *Xenopus*, *Ngnr1* (*neurogenin related 1*), the closest to mammalian *Neurog2* (Nieber et al., 2009; Osório et al., 2010), is not expressed in the Pa domain (see “preoptic area” in Wullimann et al., 2005; Osório et al., 2010), a fact likely related to the absence of Pax6 (Medina, 2008; Moreno et al., 2008, 2009; Domínguez et al., 2013). In zebrafish, only one member of the *neurogenin* family (*Neurog1*) has been identified in the pallium and prethalamic eminence but not in the Pa territory (see “preoptic area” in Wullimann and Mueller, 2004; Jeong et al., 2006; Osório et al., 2010). In agnathans, there are no data about *Ngn2*. However, as other members of the *neurogenin* family are abundantly expressed through larval stage (Guérin et al., 2009), its expression in the alar hypothalamus must not be disregarded. Together these facts suggest that the presence of *neurogenin* in the Pa territory could be an early acquisition of gnathostomes.

#### Vertebrate SPa

The expression of *Dlx*, *Arx*, and *Islet* genes in the SPa territory of vertebrates seems to be a common and early acquired trait since such expression has been documented in all vertebrates studied so far (Martínez-de-la-Torre et al., 2011; Moreno et al., 2012; Domínguez et al., 2013, 2015; Herget et al., 2014). Variability across vertebrate groups involves the co-expression, or not, with other genes such as *Nkx2.2*, *Shh*, and *Nkx2.1* in the SPa domain (Moreno and González, 2011; Moreno et al., 2012; Domínguez et al., 2013).

In lampreys, *Nkx2.1* and *Nkx2.2* are likely to co-distribute with *Dlx* in the SPa territory while not with *Hh* (homologs of *Shh*) (Myojin et al., 2001; Sugahara et al., 2011; also reviewed in Moreno and González, 2011; Domínguez et al., 2015). In zebrafish *Nkx2.1*, *Shh* and *Nkx2.2* are also likely to co-distribute without subdividing the SPa domain (Barth and Wilson, 1995; Rohr et al., 2001; van den Akker et al., 2008; Domínguez et al., 2013). However, data on zebrafish *Nkx2.1* expression must be taken with care since, recently, the orthologs previously defined as *Nkx2.1a* and *Nkx2.1b* have been renamed as *Nkx2.4b* and *Nkx2.1* respectively (Manoli et al., 2014).

Furthermore, two subcompartments have also been described in the *Dlx*-expressing alar hypothalamus of different tetrapods based on *Nkx2.2*, *Nkx2.1* and *Shh* expression (Moreno et al., 2012; Domínguez et al., 2013, 2015). One subdomain seems to co-express *Nkx2.1*, *Nkx2.2* and *Shh* while the other not. From amphibians to mammals, the domain expressing these genes seems to be almost reduced in favor of the other (Moreno et al., 2012; Domínguez et al., 2015). In amphibians and reptiles these subdomains were defined as rostro-caudal (Domínguez et al., 2015), however, a dorso-ventral interpretation was made in other vertebrates. Taking these into account, it can be concluded that in tetrapods the SPa territory is divided in dorso-ventral subdomains (SPaD and SPaV) as the updated prosomeric model proposes (Puelles et al., 2012; Domínguez et al., 2013, 2015). With all, there seems to be a tendency to the reduction of *Nkx2.2* and *Nkx2.1* from the SPa compartment in favor of the formation and or reduction of dorso-ventral compartments from fishes to mammals.

Strikingly, the data obtained in the shark apparently contradict this tendency. On one hand, only *ScNkx2.8* expression (likely to match *Nkx2.2* expression in mammals) seems to be expressed in the SPa compartment while *ScNkx2.1* and *ScShh*/Shh immunoreactivity seem to be absent, resembling a situation closer to mammals than to other vertebrate groups. In mammals, *Nkx2.1* seems to be expressed only in mantle cells of the alar hypothalamus (Shimogori et al., 2010; Puelles et al., 2012) further supporting this idea. This fact can be explained by the size of the shark pallium compared with that of other fishes. The balance among *Pax6*/*Nkx2.1* is believed to regulate the size of alar/basal compartments (van den Akker et al., 2008; Moreno et al., 2012; Puelles et al., 2012; Domínguez et al., 2013). Thus, the expanded expression of Pax6 in the shark could determine a bigger pallium over the basal hypothalamus and, consequently, a *ScNkx2.1* restriction. Noteworthy, the basal restriction of *Nkx2.1* and the co-distribution of *Dlx/Pax6* in a hypothetic SPa compartment could also be an ancestral trait as claimed by Murakami et al. (2001).

On the other hand, our data also support the existence of dorso-ventral subcompartments based on Pax6 immunoreactivity or *ScNkx2.8* co-distribution with *ScDlx2/5* but not with Sc*Shh* or Sc*Nkx2.1*. This fact raises the possibility that dorso-ventral compartments could also be addressed in fishes.

Moreover, as in shark, a population of *Pax6*-positive cells restricted to the PSPa compartment and continuous into the hypothalamus and the diencephalon has also been described in mouse (Stoykova et al., 1996). Populations of Pax6-positive cells have also been described in zebrafish under other prosomeric conceptions (Wullimann and Rink, 2001; Wullimann and Mueller, 2004) that could also support a rostro-caudal regionalization of the SPa territory in bony fishes. Together this data suggest that the presence of these cells is a conserved trait in vertebrates.

## Conclusion

Our present data suggest the existence of molecular rostro-caudal and dorso-ventral subdivisions in the alar hypothalamus of the shark equivalent to those proposed by the prosomeric model and observed in the mouse. A detailed comparative review of data among different vertebrates reveals a striking degree of conservation for the genes studied here, although there are minimal differences, which involve the phenotype of the compartments but not their organization. Concerning the shark ABB, it may be defined by the alar or basal expression of *ScDlx2/5* or *ScNkx2.1*, respectively. Besides, *ScNkx2.8*-expression overlaps over the ABB as markers like *Nkx2.2* does in other vertebrates. This revisited comparative analysis also supports that a Pa compartment positive for *Otp*, *Neurog2*, *Pax6*, *Lhx5* and a SPa territory positive for *Dlx*, *Nkx2.8*/*Nkx2.2* were already present before the agnathan-gnathostome transition.

## Author Contributions

GS-D, IR-M, EC designed the study; SM contributed to data acquisition; GS-D, AM, IQ-U, SF-G performed the experiments; GS-D, SM, IR-M, EC analyzed the data; GS-D wrote the manuscript with inputs from all authors.

## Conflict of Interest Statement

The authors declare that the research was conducted in the absence of any commercial or financial relationships that could be construed as a potential conflict of interest.

## Supplementary Material

**FIGURE S1 | Early expression of *ScNkx2.8* in whole-mounts and sections.** At stage 18 *ScNkx2.8* is expressed in a longitudinal band that spreads caudally from the rostral-most point of the neural tube without reaching the spinal cord **(A)**. The expression is more intense in a restricted domain ventrally to the optic stalk (arrowhead in **A**). This intense domain closely resembles that of *ScNkx2.*1 at the same developmental stage (see Figure 3J in Santos-Durán et al., 2015). This pattern is fairly maintained at stage 21, but in the rostral prosencephalon, *ScNkx2.8* expands ventrally in the prospective territory of the basal hypothalamus **(B)**. The expression is extended through the whole basal hypothalamus except in its caudal and ventral-most portion, a pattern that also closely resembles that of *ScNkx2.1* at early stages of development (see also Figure 4K in Santos-Durán et al., 2015). At this stage, the expression expands up to the spinal cord **(B)**. At stage 23 the expression is downregulated in the basal hypothalamus except in a rostral subdomain (black arrowhead in **C**). Of note, the expression observed before in the longitudinal axis is maintained, and is continuous with the rostral subdomain **(C)**. Besides, *ScNkx2.8* expression is slightly downregulated in a restricted territory rostral to the diencephalon (white arrowhead in **C**). Noteworthy, *ScShh* is also downregulated at this point at the same developmental stage (see Figures 3E,F in Santos-Durán et al., 2015). At stage 25, the expression of *ScNkx2.8* combined with DCX immunoreactivity reveals a co-distribution of *ScNkx2.8*-expressing cells with pioneering tracts as the postoptic commissure tract (TPOC). On transverse sections this tracts are observed as round dots at the marginal zone of *ScNkx2.8* domains (see **D** and white arrowhead in **D′**). At this point, TPOC has been observed in *S. canicula*, which course at the convergence of the alar and basal plates (see Santos-Durán et al., 2015). For abbreviations, see list.

**FIGURE S2 | Loss of *ScNkx2.8* early expression following cyclopamine treatment.** Lateral view of the cephalic region of stage 18 catshark embryos following whole-mount *in situ* hybridization (ISH) with the *ScNkx2.8* probe. Control **(A)** and cyclopamine-treated **(B)** embryos as described in Section “Materials and Methods.” The number (n) of embryos used is indicated in each case. The signal is lost in the latter.

## Abbreviations

ABB: alar-basal boundary
Ac: acroterminal region
AHy: alar hypothalamus
ap2: prosomere 2, alar part
ap3: prosomere 3, alar part
BHy: basal hypothalamus
D: diencephalon
F: forebrain
HDB: hypothalamo-diencephalic border
hp1: prosomere hp1 or peduncular
hp2: prosomere hp2 or terminal
IHB: intrahypothalamic border
M: mesencephalon
m: mantle expression
mfb: medial forebrain bundle
MM: mamillary area
Mz: mantle zone
os: optic stalk
p3: prosomere 3
p3Tg: tegmental part of prosomere 3
Pa: paraventricular area
PBHy: peduncular basal hypothalamus
PM: perimamilary area
PPa: paraventricular area, peduncular part
PRM: periretromamillary area
PSPa: subparaventricular area, peduncular part
PSPaD: subparaventricular area, peduncular and dorsal part
PSPaV: subparaventricular area, peduncular and ventral part
PThE: prethalamic eminence (ap3)
R: rhombencephalon
RM: retromamillary region
RTu: retrotuberal region
sc: spinal cord
SIBHy: basal hypothalamus, subliminal part
SIPBHy: basal hypothalamus, subliminal and peduncular part
SITBHy: basal hypothalamus, subliminal and terminal part
SOT: supraoptic tract
Sp: subpallium
SPa: subparaventricular region
SPaD: subparaventricular region, dorsal part
SPaV: subparaventricular region, ventral part
T: telencephlalon
TBHy: basal hypothalamus, terminal part
TPa: paraventricular region, terminal part
TPOC: tracts of the postoptic commissure
TSPa: subparaventricular area, terminal part
TSPaD: subparaventricular area, terminal and dorsal part
TSPaV: subparaventricular area, terminal and ventral part
Tu: tuberal region
Vz: ventricular zone
