# Supplementary figures and images for: The Shark Alar Hypothalamus: Molecular Characterization of Prosomeric Subdivisions and Evolutionary Trends
Source: Front Neuroanat. 2016 Nov 24;10:113. doi: 10.3389/fnana.2016.00113 (PMC5121248; doi:10.3389/fnana.2016.00113)

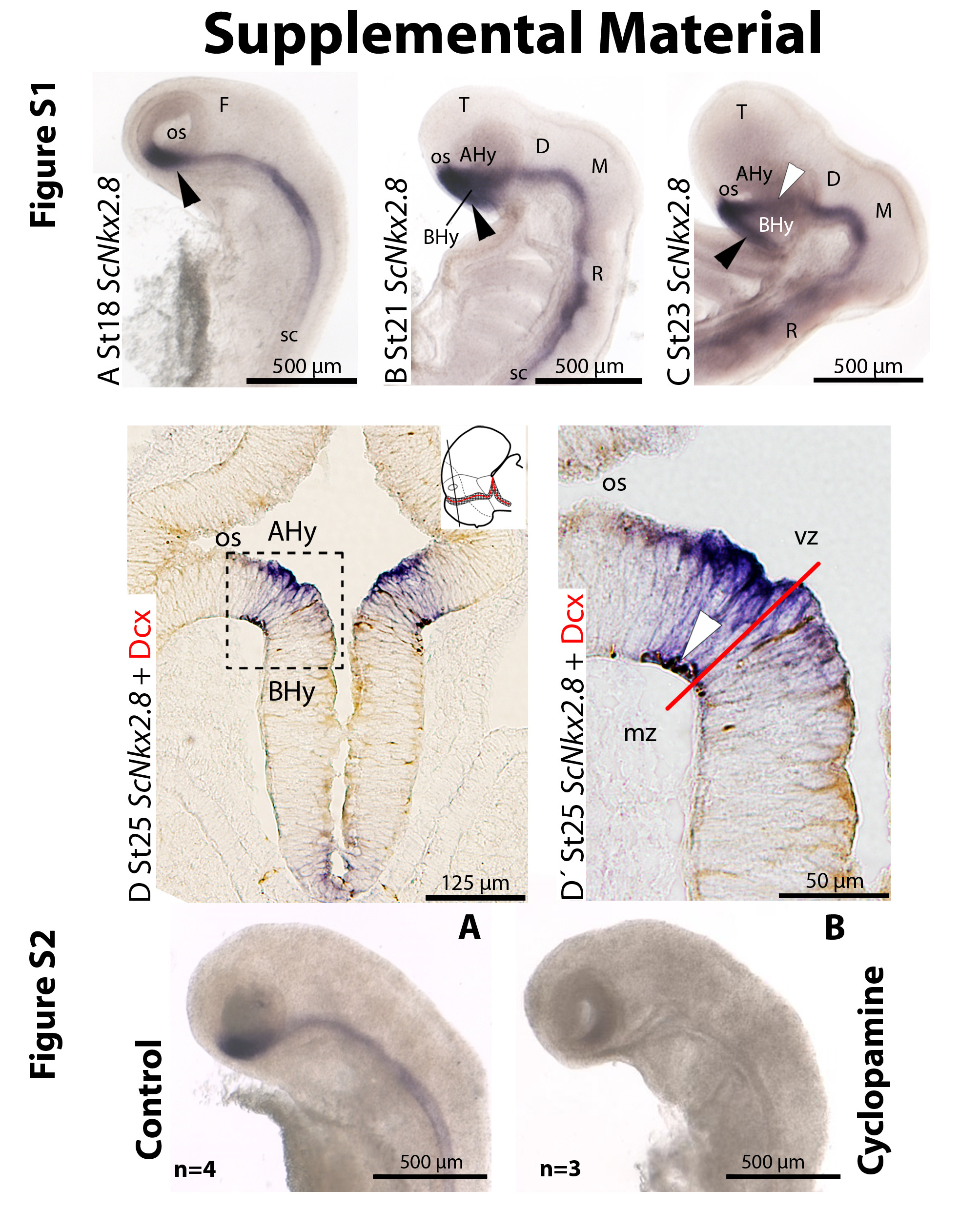

Supplement: Supplementary file 1 [file Image_1.JPEG]
